# Breastfeeding and sucking habits in children enrolled in a mother-child health program

**DOI:** 10.1186/1756-0500-7-362

**Published:** 2014-06-14

**Authors:** Teresinha Soares Pereira Lopes, Lúcia de Fátima Almeida de Deus Moura, Maria Cecília Marconi Pinheiro Lima

**Affiliations:** 1 Division of Pediatric Dentistry, Department of Pathology and Dental Clinic, Federal University of Piauí, 64049-161 Teresina, PI, Brazil; 2 Department of Human Development and Rehabilitation, Faculty of Medical Sciences, State University of Campinas, Campinas, SP, Brazil

**Keywords:** Breastfeeding, Habits, Stomatognathic system, Maternal-child health services

## Abstract

**Background:**

Early weaning can cause changes in posture and strength of the phonoarticulatory organs, favoring the installation of undesirable oral habits. The objective of the research was to evaluate the relationship between the practice of exclusive breastfeeding and its influence on the development of deleterious oral habits in children. This was a cross sectional observational study with 252 children of both sexes, between 30 and 48 months of age, attending a program of dental care for mothers and newborns. As an instrument of data collection was a questionnaire semistructured mothers of children with questions about the form and duration of breastfeeding and non-nutritive oral habits in children.

**Results:**

In this sample, 48.4% of the children were exclusively breastfed for six months; 20.2% exhibited sucking habits involving the use of a pacifier, which was more frequent among the girls. As factors associated with the decreasing of the occurrence of non-nutritive sucking habits, are a longer exclusive breastfeeding, predominant breastfeeding and breastfeeding. Children who were breastfed for six months until twelve months in an exclusive way decreased by 69.0% chances of coming to have non-nutritive sucking habits when compared with those who were breastfed up to one month.

**Conclusion:**

The longer the duration of breastfeeding, that is, exclusive, predominant or breastfeeding, the lower are the chances of children develop non-nutritive sucking habits.

## Background

The infant’s action of sucking his or her mother’s breasts favors the balance between the internal and external restraint forces of the face musculature, thus allowing for appropriate development of the stomatognathic system [1-4]. Early weaning might hinder appropriate oral motor development and cause alterations in the posture and strength of the phonatoryarticulatory organs, thus impairing the functions of chewing, swallowing, breathing and speech/sound articulation [5-7]. Therefore, inappropriate fulfillment of the urge to suckle might be related to the establishment of undesirable oral habits, such as finger sucking or the use of pacifiers or other objects to achieve satisfaction [5,7,8].

Exclusive breastfeeding (EBF) during the first months of life exerts positive influences on newborns’ nutritional, immune, emotional, and socioeconomic statuses and thus represents a crucial factor for babies’ health, with important implications for their mothers’ health [9,10]. The mother’s milk is most beneficial to infants relative to other food sources because it is better absorbed by the digestive tract, is less frequently associated with nutritional allergies, and promotes a more pleasurable development of the mother-child affective relationship [5,11].

The theoretical focus of the present study was children who were cared for under the Preventive Program for Pregnant Women and Babies, which is an outreach program that was developed by the Federal University of Piauí, with activities conducted at the Institute of Social Perinatology of Piauí. The program emphasizes issues related to breastfeeding (BF) and besides that, intends to motivate mothers to develop habits for preventing and controlling the progression of plaque-induced diseases giving guidelines to avoid the ingestion of sucrose, supervising brushing, topical fluoride application and prevention of non-nutritive sucking habits [12].

The hypothesis guiding the present study was that infants who are exclusively breastfed only for a short period or who are not breastfed at all, exhibit a higher probability of developing non-nutritive sucking habits compared with infants who are exclusively breastfed for at least six months. The aim of the present study was to associate the breastfeeding categories with harmful sucking habits in complete deciduous dentition in children enrolled in the Preventive Program for Pregnant Women and Babies.

## Methods

**Figure 1:**
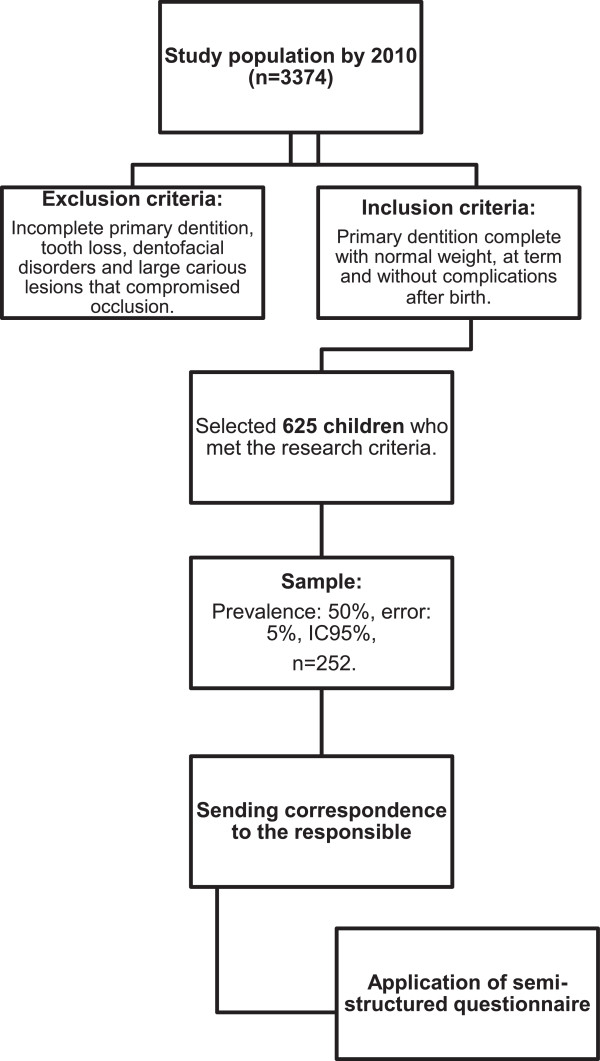
Flowchart of the study.

The present investigation was an observational, cross-sectional, and descriptive-analytic study of children aged 30 to 48 months who were cared for at a mother-child dental program, inserted in a public hospital recognized with the seal of the Baby Friendly Hospital Initiative (BFHI) in Teresina, Piauí.The study encompassed 3,374 clinical records of children who were assisted by the Preventive Program for Pregnant Women and Babies until January 2010; 625 children who met the study criteria were selected. Children were considered eligible for the study if they exhibited complete deciduous dentition, normal birth weight (equal to or higher than 2,500 g), full-term birth (more than 37 weeks of pregnancy), and a state of good health, i.e., without intercurrent events following delivery. Children with incomplete deciduous dentition, tooth loss, dentofacial anomalies, or large carious lesions affecting occlusion were excluded from the study. In addition, patients were excluded from the study if they presented syndromes, neurological diseases, or cleft lip/palate or were admitted to the neonatal intensive care unit (NICU) (Figure 1).

To calculate the sample was considered an incidence of 50% and the error was 5% with the desired precision around the prevalence, to enable confidence interval of 95%. Calculations were performed using the Epi Info version 6.04b module STATCALC software, which employs the equation s = [p(1-p)]*z^2^/d^2^, where p is the prevalence in the population; z is the percentile of the standardized normal distribution; and d is the maximum amplitude of the absolute value of the difference between the estimated and population values, adjusted by a correction factor for finite populations. Thus, the ideal representative sample for the present study was calculated in 252 children.

Letters were sent to the children’s parents and/or guardians inviting them to bring their children for an oral health assessment at the Prevent Program for Pregnant Women and Babies premises. The assessments were made in the months of April 2010 to June 2011.

The study was approved by the Research Ethics Committee of Federal University of Piauí (no. 0039.0.045.000-10). The study was conducted in compliance with Resolution no. 196/96 of the National Health Council/Ministry of Health and the Declaration of Helsinki, which regulate the guidelines and norms for research in human beings. The participants’ legal guardians signed an Informed Consent Form (ICF).

A semi-structured questionnaire, which included open and closed-ended questions, was administered to the children’s mothers to characterize the population, such as: sex, age, education of father and mother, family income, weight, number of consultations to the Prevent Program for Pregnant Women and Babies, type of breastfeeding, duration of breastfeeding, presence of oral non-nutritive sucking habits, type of habit and habits developed early in children within the family. The questionnaire structure was based on a literature review and was adapted to the study’s aims [5].

The questionnaire was pretested with 20 mothers who did not participate in the study to perform the adjustments that were necessary to improve the understanding of the investigated subjects. A pediatric dentist, responsible for the study, administered all the questionnaires.

The following categories of breastfeeding, recommended by the World Health Organization (WHO), were employed [13].

• Exclusive Breastfeeding: (EBF) when the infant is fed the mother’s milk directly or following expression, and no other liquid or solid food is administered, except for medicine drops or vitamins.

• Predominant Breastfeeding (PBF): when the infant is fed with breast milk supplemented only with water (sweetened or not), teas, other infusions and fruit juice.

• Breastfeeding (BF): when the infant remains in nonexclusive breastfeeding and not predominant, i.e., child is fed with breast milk associated with any type of supplement semisolid or solid or other milks.

• Bottle-feeding: when the children receive liquid or semi-solid food from a bottle with a nipple/teat.

A database using Epi-Info software, version 6.04, were organized with double entry, in order to check errors thus ensuring a better quality of information. The data were imported, processed, and analyzed by the SPSS® version 18.0 software for Windows. The odds ratio (OR) was used as the size measure in the bivariate analysis, with a 95% confidence interval (95% CI). To test the correlation between the presence of non-nutritive sucking habits and the independent variables, Pearson’s chi-square (χ^2^) test was used, and p values ≤ 0.05 were considered to be significant.

To study the effect of the control on the dependent variables, a conceptual hierarchical model was elaborated in which the socioeconomic variables were placed in the first level and the BF-related variables were placed in the second level. In this method of analysis, the variables are controlled for all of the other variables at the same and/or upper level. Variables with p ≤ 0.02 upon bivariate analysis were included in the model. Unconditional multiple logistic regression was used in the analysis (adjusted for the possible confounding variables), and the model adjustment was analyzed by the Hosmer-Lemeshow test. The 95% CIs were calculated, and correlations with p < 0.05 were considered to be statistically significant.

## Results

The profile of the investigated sample is described in Table 1.

**Table 1:** Sample profile

**Variable**	**N**	**%**
**Sex**		
Male	139	55.2
Female	113	44.8
**Age range (months)**		
30 to 36	73	28.9
37 to 42	132	52.4
43 to 48	47	18.7
**Birth weight (grams)**		
2,500 – 3,500	133	52.9
3,501 – 4,500	115	45.6
4,501 – 5,000	04	1.5
**Family income (minimum wage)**		
≤ 1	99	39.3
2 – 3	114	45.2
≥ 3	39	15.5
**Caregiver**		
Mother	233	92.5
Father	08	3.2
Other	11	4.3
**Number of visits in the program**		
1 – 3	178	70.6
4 – 6	52	20.6
7 – 9	22	8.8
**Exclusive breastfeeding (months)**		
< 1	34	13.5
2-3	39	15.5
4-5	57	22.6
≥ 6	122	48.4
**Predominant breastfeeding (months)**		
< 1	11	4.4
2 – 3	17	6.7
4 – 5	30	11.9
≥ 6	194	77.0
**Breastfeeding (months)**		
1-6	43	17.1
7 – 12	100	39.7
13 – 24	56	22.2
> 24	53	21.0

Of the 252 children, 183 (72.7%) did not exhibit non-nutritive oral habits such as finger sucking or the use of a pacifier. Of the 69 (27.3%) children who exhibited non-nutritive oral habits, 51 (20.2%) used pacifiers, and 18 (7.1%) exhibited finger sucking. Regarding the use of bottle, 70 (27.8%) of the children used this instrument to be fed, with a mean of 5.2 months, standard deviation of 1 month and a maximum usage time of 14 months (Table 2).

**Table 2:** Distribution by types of non-nutritive and nutritive sucking habits

**Types of non-nutritive and nutritive sucking habits**	**Present**		**Total**
	**Yes**	**No**	**n (%)**
	**n (%)**	**n (%)**	
**Digital**	18 (7,1)	234 (92,9)	252 (100)
**Pacifier**	51 (20,2)	201 (79,8)	252 (100)
**Bottle**	70 (27,8)	182 (72,2)	252 (100)

Upon bivariate analysis (described in Tables 3 and 4), the presence of harmful habits was associated with the characteristics of the investigated sample, and the results indicated a higher prevalence of non-nutritive sucking habits among the females (33.6%) compared to male and a decrease of this kind of habit as longer the time of exclusive breastfeeding (p = 0,007) and breastfeeding (p < 0,001) of children.

**Table 3:** Prevalence (%) and odds ratio (OR) for association study of socioeconomic characteristics with non-nutritive sucking habits

**Variable**	**Non-nutritive sucking habits**			**Raw OR**	**95% CI**	**p**^**a**^
	**Yes**	**No**	**Total**			
	**n (%)**	**n (%)**	**N**			
**Sex**						0.045*
Male	31 (22.3)	108 (77.7)	139	1		
Female	38 (33.6)	75 (66.4)	113	1.77	1.01-3.09	
**Age range**						0.959
Up to 36 months	16 (27.1)	43 (72.9)	59	1		
37-48 months	53 (27.5)	140 (72.5)	193	1.02	0.53-1.96	
**Father’s educational level**						0.128
Elementary	11 (30.6)	25 (69.4)	36	1		
Secondary	53 (25.6)	154 (74.4)	207	0.35	0.08-1.57	
Higher	05 (55.6)	04 (44.4)	09	0.28	0.07-1.06	
**Mother’s educational level**						0.392
Elementary	16 (32.7)	33 (67.3)	49	1		
Secondary	49 (25.4)	144 (74.6)	193	0.65	0.35-1.24	
Higher	04 (40.0)	06 (60.0)	10	1.81	0.49-6.64	
**Family income**						0.156
1 minimum wage	22 (22.2)	77 (77.8)	99	1		
2-3 minimum wage	38 (33.3)	76 (66.7)	114	0.95	0.39-2.30	
> 3	09 (23.1)	30 (76.9)	39	0.60	0.26-1.39	

Educational level: Elementary education corresponds to zero to eight years of schooling. Secondary education corresponds to nine to 11 years of schooling. Higher education corresponds to 12 or more years of schooling.

95% CI = confidence interval; OR = odds ratio.

^a^Pearson’s chi-square (χ^2^), *significant.

**Table 4:** Prevalence (%) and odds ratio (OR) to study the association of breastfeeding with non-nutritive sucking habits

**Variable**	**Non-nutritive sucking habits**			**OR**	**95% CI**	**p**^**a**^
	**Yes**	**No**	**Total**			
	**N (%)**	**N (%)**	**N**			
**Exclusive breastfeeding**						0.007*
≤ 1 month	15 (44.1)	19 (55.9)	34	1		
2-3 months	14 (35.9)	25 (64.1)	39	1.61	0.78-3.31	
4-5 months	18 (31.6)	39 (68.4)	57	1.30	0.68-2.48	
6-12 months	22 (18.0)	100 (82.0)	122	0.39	0.22-0.70	
**Predominant breastfeeding**						0.065
≤ 1 month	06 (54.5)	05 (45.5)	11	1		
2-3 months	08 (47.1)	09 (52.9)	17	2.53	0.94-6.89	
4-5 months	09 (30.0)	21 (70.0)	30	1.16	0.50-2.67	
6-12 months	12 (22.6)	41 (77.4)	53	0.73	0.36-1.49	
≥12 months	34 (24.1)	100 (75.9)	141	0.70	0.40-1.20	
**Breastfeeding**						<0.001*
1-6 months	27 (62.8)	16 (37.2)	43	1		
7 – 12 months	23 (23.0)	77 (77.0)	100	0.69	0.38-1.23	
13 – 24 months	12 (21.4)	44 (78.6)	56	0.66	0.32-1.35	
> 24 months	07 (13.2)	46 (88.8)	33	0.12	0.14-0.79	

95% CI = confidence interval; OR = odds ratio.

^a^Pearson’s chi-square (χ^2^), *significant.

A multivariate analysis based on the hierarchical levels (Table 5) revealed that the factors that remained associated with the presence of non-nutritive sucking habits were the sex (p = 0.011), EBF (p = 0.001), PBF (p = 0,011) and BF (p < 0.001). The female has a risk factor for non-nutritious habits when compared to male (OR = 2,15; IC95% : 1,19 -3,88), an increased exclusive breastfeeding for 6–12 months (OR = 0,31; IC95% : 0,13 -0,73), predominant breastfeeding for 6–12 months (OR = 0.31; IC95%:0.17-0.84) and 12 months (OR = 0,37; IC95%: 0,16-0,87), and breastfeeding were associated factors, decreasing the chances of non-nutritive sucking habits in the studied group.

**Table 5:** Logistic regression model for non-nutritive sucking habits

**Variable**	**Raw OR**	**95% CI**	**p**^**a**^	**Adjusted OR**	**95% CI**	**p**^**b**^
**Level I**						
**Genre**			0.045*			0.011*
Male	1			1		
Female	1.77	1.01-3.09		2.15	1.19-3.88	
**Level II**						
**Exclusive breasfeeding**			0,007*			0.001*
≤ 1 month	1			1		
2-3 months	1.61	0.78-3.31		0.73	0.28-1.90	
4-5 months	1.30	0.68-2.48		0.70	0.28-1.73	
6-12 meses	0.39	0.22-0.70		0.31	0.13-0.73	
**Predominant breastfeeding**			0,065			0.011*
≤ 1 month	1			1		
2-3 months	2.53	0.94-6.89		1.70	0.53-5.39	
4-5 months	1.16	0.50-2.67		0.44	0.15-1.30	
6-12 months	0.73	0.36-1.49		0.31	0,17-0.84	
≥ 12 months	0.70	0.40-1.20		0.37	0.16-0.87	
**Breastfeeding**			<0,001*			<0,001*
1-6 months	1			1		
7-12 months	0.69	0.38-1.23		0.20	0.09-0.48	
13-24 months	0.66	0.32-1.35		0.18	0.07-0.48	
> 24 months	0.12	0.14-0.79		0,10	0.04-0.31	

LeveI I: adjusted to the father’s educational level + the family income; Level II: adjusted to Level I + Level II; ^a^Pearson’s chi-square (χ^2^), ^b^Wald’s test and *significant; *Hosmer-Lemeshow* test, p = 0.402.

## Discussion

The practice of breastfeeding (BF) meets both the physical and psychological needs of newborns and is crucial for development of the normal sucking patterns [14] that are necessary for promoting and protecting the children’s health [3,8]. However, breast-feeding is not practiced as much as is necessary to ensure an appropriate nutritional state in children and thus reduce the infant morbimortality worldwide [15,16].

The present study revealed that all of the mothers started BF during the newborns’ first hours of life, as the sample consisted of full-term newborns who had normal birth weights and good states of health (Table 1) and who were born at a public maternity hospital that adopted the ten-step program for successful BF, which forbids the use of artificial nipples and bottles during the hospital stay according to the recommendations of the World Health Organization [17].

Encouraging the practice of breastfeeding depends on motivational strategies used in the services such as maternities, child care centers and institutes of perinatology, but it has been observed that breastfeeding is more emphasized in public maternities than in private facilities [16,18]. The lack of motivation of mothers about the benefits of breastfeeding is one of the risk factors for weaning [6,15] because they often disrupt breastfeeding due to insecurity or lack of interest in implementing this practice. However, there are other factors that can contribute to early weaning such as a lower maternal age, socioeconomic status, less education level, maternal employment, conditions of delivery, neonate weight at birth and the use of pacifier [2,9,10].

In the present study, we found a satisfactory adherence to the practice of breastfeeding, with the rate of EBF up to six months of the child’s age (Table 4), very close to rate considered good for WHO [19]. Besides, more than two thirds of the children prolonged breastfeeding over 24 months old. Other studies have found EBF rates ranging from 1.8% to 48.3% [20,21], moreover a longitudinal study reported that found no child who has been breastfed exclusively until six months of age [11].

Starting in the 1990s, the rate of BF has been increasing in both developed and underdeveloping countries; however, this is not the case with EBF. For example, in Africa, the rate of BF up to 11 months is 90%, whereas the rate of EBF up to six months is 25%; in Bolivia, the rate of BF up to 12 months is 85%, whereas and the rate of EBF up to six months is 20 - 25%; in Pakistan, the rate of BF up to 12 months is 88%, whereas the rate of and EBF up to six months is 10%; in the United States, the rate of BF up to 12 months is 16.1%, whereas the rate of EBF up to six months is 13.3% [15].

In Brazil, according to Study II on the Prevalence of Breastfeeding [22], the median of EBF is 1.8 months, which is higher than the median of one month found in the previous study, thus indicating an increase in the BF rate. Nonetheless, according to the 2009 study [22], the prevalence of EFB among infants younger than six months old was 41.0% at the national level and 43.7% in Teresina (PI), whereby the latter was higher than the national average and the rates in this study was higher.

Sucking not only is a means of nutrition for newborns but also satisfies a natural desire because the mouth represents the first source of pleasure and of communication with the world [2,8]. The sucking reflex appears during the 32nd week of intrauterine life, which makes fetuses neurologically fit for BF [23]. Therefore, healthy newborns exhibit an inherent biological tendency to suck, which is considered to be normal until the age up to 24 months [24]. When this tendency is not fully satisfied via BF, the infants seek other sources of satisfaction [25] and thus develop harmful oral habits, such as sucking the fingers or the tongue, sucking and biting the lips, atypical deglutition, onychophagia, and sucking pacifiers or other objects [8,25].

Non-nutritive sucking habits were present in the sample studied, corroborating other studies [2,7,8,25-27], however, with the lowest rates observed in previous studies, possibly reflecting the high rate of breastfeeding (BF), predominant breastfeeding (PBF) and exclusive breastfeeding (EBF). Higher prevalence of pacifier-sucking habit (Table 2) was observed, the same result was observed in other studies [11,28,29]. National survey showed that in the northeastern area in Brazil the habit of pacifier sucking was considered high (42,6%), whereas in Teresina (PI) this percentage was lower (29,7%), but still higher than the data found in the present study (20,2%). The differences found in the studies may be explained in part by the origin of the sample, in addition to the fact that, in these study, the mothers attended a maternity hospital with the seal of BFHI [29], and are also assisted by a maternal and child dental care program, the Preventive Program for Pregnant Women and Babies, that in addition to encouraging breastfeeding, motivates the adoption of healthy mothers habits [12].

In our research, some children used bottle to be fed besides all of them had been initially breastfed. It was also observed that the average time for using the bottle was five months and that many children only did so for a period of one month. This fact shows that many mothers feel insecure to promote a partial weaning, and for practical and cultural issues they decide to use the bottle to support for weaning [30]. It is usual to find mothers who are enrolled in the Prevent Program for Pregnant Women and Babies reporting that their children after being exclusively breastfed for a longer period of time, do not accept pacifier or bottle. As a result, a lower percentage of children were using pacifier or bottle, when compared to the second survey of breastfeeding, found that more than half of children took the bottle [22].

There was no correlation between the children’s age and the presence of non-nutritive oral habits in the present study, which disagrees with the results of other studies that demonstrated a significant correlation [2,24]. This finding might be partially explained by the young ages of the investigated children. The reduction of harmful oral habits parallel to the increase in age occurs partially because, upon growing, children tend to forsake the habits associated with the pleasure of sucking. Therefore, it is expected that a large fraction of children forsake their non-nutritive sucking habits at the end of the oral stage because the maintenance of these habits for longer periods might result in complex alterations of the oral motor development [8,26].

According to mother’s schooling also was not associated with the non-nutritive sucking habits like the type finger or pacifier (Table 5). The literature reports controversial data regarding this social indicator, as some studies did [21,25] and others did not [1,26] find a correlation between this variable and the presence of sucking habits.

Several investigations have demonstrated a relationship between BF and nonnutritive sucking habits [1,2,6,8,27,29], which was also found in the present study, as the children who were exclusively breastfed for more than six month exhibited a considerable and progressive reduction of the odds of developing non-nutritive oralhabits, compared with the children who were breastfed for up to one month (p = 0.001).

These results indicate that exclusive breastfeeding is an associated factor to decrease the suction by non-nutritive habits. As the same way, the predominant breastfeeding and total breastfeeding are also presented as an important factor to decrease the practice of non-nutritive habits (p = 0,011 and p < 0,001 respectively).

As a limitation of the study, we can point out the difficulty in precisely determine the isolated role of breastfeeding in the prevention of non-nutritive sucking habits by the very nature of a cross-sectional survey and also, by the characteristics of our sample. We suggest that more research be conducted in order to confirm or not our findings.

## Conclusions

Rate of exclusive breastfeeding in infants up to six months of age was high, which is higher than the national average. Maintaining the three forms of breastfeeding exerted positive influences in reducing the development of non-nutritive sucking habits. The high rates of breastfeeding and the low index of non-nutritive sucking habits found in the present study are partly due to the health promotion strategies adopted by the institution.

## Abbreviations

CI: Confidence interval; BF: Breastfeeding; BFHI: Baby friendly hospital initiative; EBF: Exclusive breastfeeding; NICU: Neonatal intensive care unit; OR: Odds ratio; PBF: Predominant breastfeeding; PI: Piauí; SPSS®: Statistical package for the social sciences; WHO: World Health Organization.

## Competing interests

The authors declare that they have no competing interests.

## Authors’ contributions

TSPL conception, design, data collection, analysis and interpretation of data, drafting and critical revision of the manuscript. LFADM participated in critical revision of the manuscript and format. MCMPL participated in data analysis, interpretation, contributed to the drafting and critical revision of the manuscript. All authors read and approved the final interpretation and drafting the manuscript.
